# *N*-(2,5-Dimeth­oxy­phen­yl)-*N*′-(4-hy­droxy­pheneth­yl)urea

**DOI:** 10.1107/S1600536810038535

**Published:** 2010-10-02

**Authors:** Hyeong Choi, Byung Hee Han, Yong Suk Shim, Sung Kwon Kang, Chang Keun Sung

**Affiliations:** a Department of Chemistry, Chungnam National University, Daejeon 305-764, Republic of Korea; b Department of Food Science and Technology, Chungnam National University, Daejeon 305-764, Republic of Korea

## Abstract

In the title compound, C_17_H_20_N_2_O_4_, the 2,5-dimeth­oxy­phenyl unit is almost planar, with an r.m.s. deviation of 0.015 Å. The dihedral angle between the 2,5-dimeth­oxy­phenyl ring and the urea plane is 20.95 (8)°. The H atoms of the urea NH groups are positioned *syn* to each other. The mol­ecular structure is stabilized by a short intra­molecular N—H⋯O hydrogen bond. In the crystal, inter­molecular N—H⋯O and O—H⋯O hydrogen bonds link the mol­ecules into a three-dimensional network.

## Related literature

For general background to tyrosinase, see: Kubo *et al.* (2000 ▶); Perez-Gilbert & Garcia-Carmona (2001 ▶). For the development of tyrosinase inhibitors, see: Shiino *et al.* (2001 ▶); Khan *et al.* (2006 ▶); Garcia & Fulrton (1996 ▶); Kojima *et al.* (1995 ▶); Cabanes *et al.* (1994 ▶); Lemic-Stojcevic *et al.* 1995 ▶); Casanola-Martin *et al.* (2006 ▶); Thanigaimalai *et al.* (2010 ▶); Passi & Nazzaro-Porro (1981 ▶).
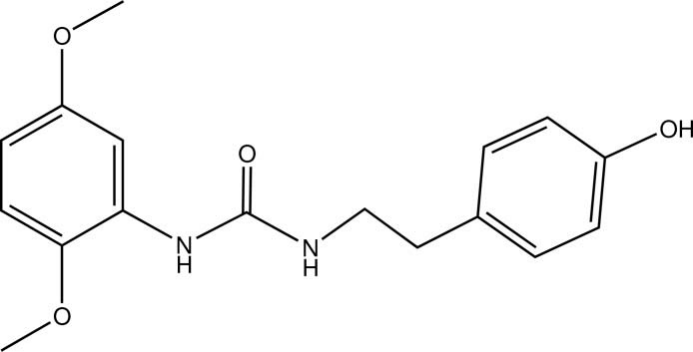


## Experimental

### 

#### Crystal data

C_17_H_20_N_2_O_4_
*M* *_r_* = 316.35
Monoclinic, 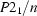

*a* = 10.7275 (6) Å
*b* = 9.6016 (5) Å
*c* = 16.9388 (10) Å
β = 107.838 (2)°
*V* = 1660.84 (16) Å^3^
*Z* = 4
Mo *K*α radiation
μ = 0.09 mm^−1^
*T* = 296 K
0.31 × 0.27 × 0.13 mm

#### Data collection

Bruker SMART CCD area-detector diffractometer
13358 measured reflections
3184 independent reflections
2296 reflections with *I* > 2σ(*I*)
*R*_int_ = 0.044

#### Refinement

*R*[*F* ^2^ > 2σ(*F* ^2^)] = 0.059
*wR*(*F* ^2^) = 0.186
*S* = 1.06
3184 reflections
218 parameters
H atoms treated by a mixture of independent and constrained refinement
Δρ_max_ = 0.32 e Å^−3^
Δρ_min_ = −0.46 e Å^−3^

### 

Data collection: *SMART* (Bruker, 2002 ▶); cell refinement: *SAINT* (Bruker, 2002 ▶); data reduction: *SAINT*; program(s) used to solve structure: *SHELXS97* (Sheldrick, 2008 ▶); program(s) used to refine structure: *SHELXL97* (Sheldrick, 2008 ▶); molecular graphics: *DIAMOND* (Brandenburg, 2010 ▶); software used to prepare material for publication: *WinGX* (Farrugia, 1999 ▶).

## Supplementary Material

Crystal structure: contains datablocks global, I. DOI: 10.1107/S1600536810038535/jh2214sup1.cif

Structure factors: contains datablocks I. DOI: 10.1107/S1600536810038535/jh2214Isup2.hkl

Additional supplementary materials:  crystallographic information; 3D view; checkCIF report

## Figures and Tables

**Table 1:** Hydrogen-bond geometry (Å, °)

*D*—H⋯*A*	*D*—H	H⋯*A*	*D*⋯*A*	*D*—H⋯*A*
N7—H7⋯O20	0.82 (3)	2.23 (2)	2.617 (3)	109 (2)
N7—H7⋯O19^i^	0.82 (3)	2.48 (3)	3.182 (3)	144 (2)
N10—H10⋯O19^i^	0.86 (3)	2.23 (3)	3.005 (3)	150 (2)
O19—H19⋯O9^ii^	0.86 (4)	1.80 (4)	2.654 (3)	172 (4)

Symmetry codes: (i) 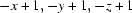; (ii) 
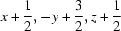
.

**Fig. 1.**
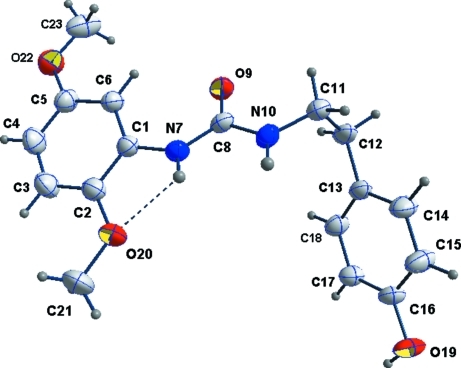
Molecular structure of (l), showing the atom-numbering scheme and 50% probability ellipsoids. Intramolecular N—H···O bond is shown as dashed lines.

**Fig. 2.**
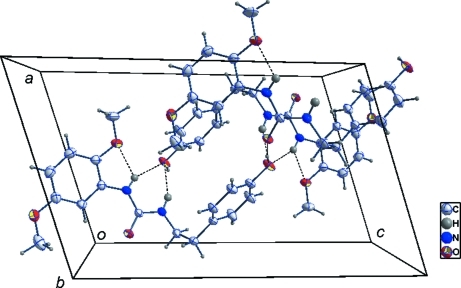
Part of the crystal structure of (I), showing 3-D network of molecules linked by intermolecular N—H···O and O—H···O hydrogen bonds (dashed lines).

## supplementary crystallographic information

### Comment

Tyrosinase known as a polyphenol oxidase, is a multifunctional copper-containing enzyme widely distributed in nature. It is the key enzyme in the undesirable browning of fruits and vegetables, and coloring of skin, hair, and eyes in animals (Kubo *et al.*, 2000; Perez-Gilbert & Garcia-Carmona, 2001). Nowadays, tyrosinase inhibitors are thought to be clinically useful for the treatment of some dermatological disorders associated with melanin hyperpigmentation (Shiino *et al.*, 2001) and useful in cosmetic products and food industry (Khan *et al.*, 2006). Recently, various tyrosinase inhibitors have been reported such as hydroquinone (Garcia & Fulrton, 1996), ascorbic acid derivatives (Kojima *et al.*, 1995), kojic acid (Cabanes *et al.*, 1994), azelaic acid (Lemic-Stojcevic *et al.*, 1995), arbutin (Casanola-Martin *et al.*, 2006) and *N*-phenylthiourea (PTU) (Thanigaimalai *et al.*, 2010). Most of the tyrosinase inhibitors are phenol/catechol derivatives, structurally similar to tyrosine or *L*-DOPA, which act as suicide substrates of tyrosinase (Passi & Nazzaro-Porro, 1981). However, most of them are not potent enough to put into practical use due to their weak individual activities or safety concerns. Undoubtedly, it is required to search and develop novel tyrosinase inhibitors with better activities together with lower side effects. In continuing our research on the development of tyrosinase inhibitors for new whitening agents, we have synthesized the title compound, (I), from the reaction of 2-(4-hydroxyphenyl)ethyl amine and 2,5-dimethoxyphenyl isocyanate under ambient condition. Here, we report the crystal structure of the title compound, (I).

The 2,5-dimethoxyphenyl moiety is almost planar with r.m.s. deviation of 0.015 Å from the corresponding least-squares plane defined by the nine constituent atoms. The dihedral angle between the phenyl ring and the plane of urea moiety is 20.95 (8) °. The molecular structure is stabilized by a short intramolecular N7—H7···O20 hydrogen bond (Fig. 1). In the crystal, intermolecular N—H···O and O—H···O hydrogen bonds link the molecules into a three-dimensional network (Fig. 2, Table 1). The H atoms of the NH groups of urea are positioned *syn* to each other.

### Experimental

The tyramine and 2,5-dimethoxyphenyl isocyanate were purchased from Sigma Chemical Co. Solvents used for organic synthesis were redistilled before use. All other chemicals and solvents were of analytical grade and used without further purification. The title compound (I) was prepared from the reaction of 2-(4-hydroxyphenyl)ethyl amine (0.20 g, 1 mmol) with 2,5-dimethoxyphenyl isocyanate (0.18 g, 1.2 mmol) in acetonitrile (8 ml) and added 4-(dimethylamino)pyridine (0.06 g, 0.5 mmol) as a catalyst, with stirring. The reaction was completed within 5 h at room temperature. The solvents were removed under reduced pressure. The solids collected and washed with dichloromethane. Removal of the solvent gave a light yellow solid (69%, m.p. 436 K). Single crystals were obtained by slow evaporation of the ethanol at room temperature.

### Refinement

The H atoms of the NH and OH groups were located in a difference Fourier map and refined freely. The remaining H atoms were positioned geometrically and refined using a riding model with C—H = 0.93–0.97 Å, and with *U*_iso_(H) = 1.2*U*_eq_ (C) for aromatic and metylene, and 1.5*U*_eq_(C) for methyl H atoms.

### Crystal data

**Table:**

C_17_H_20_N_2_O_4_	*F*(000) = 672
*M**_r_* = 316.35	*D*_x_ = 1.265 Mg m^−^^3^
Monoclinic, *P*2_1_/*n*	Mo *K*α radiation, λ = 0.71073 Å
Hall symbol: -P 2yn	Cell parameters from 5519 reflections
*a* = 10.7275 (6) Å	θ = 2.5–27.8°
*b* = 9.6016 (5) Å	µ = 0.09 mm^−^^1^
*c* = 16.9388 (10) Å	*T* = 296 K
β = 107.838 (2)°	Needle, colourless
*V* = 1660.84 (16) Å^3^	0.31 × 0.27 × 0.13 mm
*Z* = 4	

### Data collection

**Table:**

Bruker SMART CCD area-detector diffractometer	*R*_int_ = 0.044
φ and ω scans	θ_max_ = 26.0°, θ_min_ = 2.0°
13358 measured reflections	*h* = −13→6
3184 independent reflections	*k* = −11→9
2296 reflections with *I* > 2σ(*I*)	*l* = −20→16

### Refinement

**Table:**

Refinement on *F*^2^	0 restraints
Least-squares matrix: full	H atoms treated by a mixture of independent and constrained refinement
*R*[*F*^2^ > 2σ(*F*^2^)] = 0.059	*w* = 1/[σ^2^(*F*_o_^2^) + (0.0966*P*)^2^ + 0.4089*P*] where *P* = (*F*_o_^2^ + 2*F*_c_^2^)/3
*wR*(*F*^2^) = 0.186	(Δ/σ)_max_ < 0.001
*S* = 1.06	Δρ_max_ = 0.32 e Å^−^^3^
3184 reflections	Δρ_min_ = −0.46 e Å^−^^3^
218 parameters	

### Special details

**Table:**

Geometry. All e.s.d.'s (except the e.s.d. in the dihedral angle between two l.s. planes) are estimated using the full covariance matrix. The cell e.s.d.'s are taken into account individually in the estimation of e.s.d.'s in distances, angles and torsion angles; correlations between e.s.d.'s in cell parameters are only used when they are defined by crystal symmetry. An approximate (isotropic) treatment of cell e.s.d.'s is used for estimating e.s.d.'s involving l.s. planes.

### Fractional atomic coordinates and isotropic or equivalent isotropic displacement parameters (Å^2^)

**Table:**

	*x*	*y*	*z*	*U*_iso_*/*U*_eq_	
C1	0.3898 (2)	0.5854 (2)	0.12845 (14)	0.0581 (5)	
C2	0.5212 (2)	0.6136 (3)	0.13837 (16)	0.0698 (7)	
C3	0.5565 (3)	0.6644 (3)	0.0720 (2)	0.0856 (8)	
H3	0.644	0.6835	0.0783	0.103*	
C4	0.4643 (3)	0.6869 (3)	−0.00277 (19)	0.0845 (8)	
H4	0.4896	0.7201	−0.0471	0.101*	
C5	0.3347 (3)	0.6610 (3)	−0.01298 (15)	0.0724 (7)	
C6	0.2967 (2)	0.6085 (2)	0.05238 (14)	0.0636 (6)	
H6	0.209	0.589	0.0452	0.076*	
N7	0.35963 (18)	0.5297 (2)	0.19699 (12)	0.0639 (5)	
H7	0.423 (3)	0.498 (3)	0.2325 (16)	0.068 (7)*	
C8	0.24229 (19)	0.5317 (2)	0.21258 (12)	0.0540 (5)	
O9	0.14248 (14)	0.58188 (19)	0.16417 (9)	0.0701 (5)	
N10	0.24242 (19)	0.4718 (2)	0.28412 (11)	0.0638 (5)	
H10	0.315 (3)	0.441 (3)	0.3168 (16)	0.075 (8)*	
C11	0.1271 (2)	0.4629 (3)	0.31035 (13)	0.0683 (7)	
H11A	0.1342	0.3814	0.3453	0.082*	
H11B	0.0514	0.4501	0.2618	0.082*	
C12	0.1046 (2)	0.5896 (3)	0.35756 (13)	0.0684 (7)	
H12A	0.0949	0.6706	0.322	0.082*	
H12B	0.0231	0.5774	0.3703	0.082*	
C13	0.21277 (19)	0.6172 (2)	0.43716 (13)	0.0558 (5)	
C14	0.2326 (3)	0.5296 (3)	0.50382 (15)	0.0820 (8)	
H14	0.1792	0.4519	0.4992	0.098*	
C15	0.3299 (3)	0.5540 (3)	0.57772 (16)	0.0874 (9)	
H15	0.3413	0.4926	0.6219	0.105*	
C16	0.40944 (19)	0.6679 (2)	0.58626 (13)	0.0597 (6)	
C17	0.3892 (2)	0.7585 (2)	0.52187 (14)	0.0638 (6)	
H17	0.4404	0.8383	0.5274	0.077*	
C18	0.2917 (2)	0.7315 (2)	0.44770 (14)	0.0654 (6)	
H18	0.2798	0.7935	0.4038	0.078*	
O19	0.50449 (17)	0.6876 (2)	0.66089 (11)	0.0816 (6)	
H19	0.552 (3)	0.759 (4)	0.658 (2)	0.122*	
O20	0.60539 (16)	0.5880 (3)	0.21599 (12)	0.0954 (7)	
C21	0.7414 (3)	0.6140 (7)	0.2294 (2)	0.1518 (19)	
H21A	0.7898	0.5914	0.2857	0.228*	
H21B	0.7541	0.7105	0.2192	0.228*	
H21C	0.7719	0.5574	0.1923	0.228*	
O22	0.2495 (2)	0.6902 (3)	−0.08960 (12)	0.0991 (7)	
C23	0.1155 (4)	0.6852 (3)	−0.10046 (19)	0.1056 (11)	
H23A	0.0681	0.7078	−0.1569	0.158*	
H23B	0.094	0.7512	−0.064	0.158*	
H23C	0.0919	0.5933	−0.0879	0.158*	

### Atomic displacement parameters (Å^2^)

**Table:**

	*U*^11^	*U*^22^	*U*^33^	*U*^12^	*U*^13^	*U*^23^
C1	0.0566 (11)	0.0614 (13)	0.0551 (12)	0.0002 (9)	0.0154 (10)	−0.0068 (10)
C2	0.0567 (13)	0.0777 (16)	0.0735 (15)	−0.0004 (11)	0.0175 (12)	−0.0106 (12)
C3	0.0665 (15)	0.097 (2)	0.100 (2)	−0.0084 (14)	0.0355 (15)	−0.0010 (17)
C4	0.0879 (18)	0.0901 (19)	0.0855 (19)	−0.0109 (15)	0.0411 (16)	0.0065 (15)
C5	0.0828 (16)	0.0724 (16)	0.0603 (14)	−0.0089 (12)	0.0195 (12)	0.0028 (11)
C6	0.0610 (12)	0.0702 (15)	0.0565 (13)	−0.0088 (10)	0.0135 (10)	−0.0019 (11)
N7	0.0488 (10)	0.0852 (14)	0.0512 (11)	0.0095 (9)	0.0059 (8)	0.0050 (9)
C8	0.0531 (11)	0.0588 (12)	0.0426 (10)	0.0045 (9)	0.0037 (9)	−0.0033 (9)
O9	0.0538 (8)	0.0941 (12)	0.0551 (9)	0.0151 (8)	0.0057 (7)	0.0144 (8)
N10	0.0603 (11)	0.0815 (13)	0.0453 (10)	0.0137 (9)	0.0100 (8)	0.0071 (9)
C11	0.0644 (13)	0.0858 (17)	0.0483 (12)	−0.0152 (11)	0.0079 (10)	−0.0045 (11)
C12	0.0465 (11)	0.1008 (18)	0.0511 (12)	0.0057 (11)	0.0050 (9)	−0.0050 (12)
C13	0.0456 (10)	0.0698 (14)	0.0466 (11)	0.0055 (9)	0.0061 (8)	−0.0026 (10)
C14	0.0843 (17)	0.0838 (17)	0.0614 (14)	−0.0297 (14)	−0.0020 (12)	0.0050 (13)
C15	0.1003 (19)	0.0795 (17)	0.0587 (14)	−0.0219 (15)	−0.0109 (13)	0.0176 (13)
C16	0.0501 (11)	0.0611 (13)	0.0541 (12)	0.0033 (9)	−0.0044 (9)	0.0008 (10)
C17	0.0597 (12)	0.0574 (12)	0.0646 (13)	−0.0045 (10)	0.0047 (10)	0.0023 (10)
C18	0.0679 (14)	0.0651 (14)	0.0544 (12)	0.0060 (11)	0.0056 (10)	0.0120 (10)
O19	0.0738 (11)	0.0760 (12)	0.0659 (10)	−0.0059 (8)	−0.0214 (8)	0.0041 (8)
O20	0.0496 (9)	0.1491 (19)	0.0801 (12)	0.0044 (10)	0.0088 (8)	−0.0044 (12)
C21	0.0506 (16)	0.283 (6)	0.114 (3)	−0.004 (2)	0.0139 (17)	−0.020 (3)
O22	0.0948 (14)	0.1328 (18)	0.0650 (11)	−0.0083 (12)	0.0173 (10)	0.0177 (11)
C23	0.136 (3)	0.073	0.0813 (19)	−0.0271 (17)	−0.0063 (19)	0.0146 (15)

### Geometric parameters (Å, °)

**Table:**

C1—C6	1.385 (3)	C12—H12A	0.97
C1—C2	1.394 (3)	C12—H12B	0.97
C1—N7	1.403 (3)	C13—C18	1.364 (3)
C2—O20	1.370 (3)	C13—C14	1.371 (3)
C2—C3	1.381 (4)	C14—C15	1.382 (3)
C3—C4	1.364 (4)	C14—H14	0.93
C3—H3	0.93	C15—C16	1.367 (3)
C4—C5	1.370 (4)	C15—H15	0.93
C4—H4	0.93	C16—C17	1.360 (3)
C5—O22	1.368 (3)	C16—O19	1.373 (2)
C5—C6	1.387 (3)	C17—C18	1.391 (3)
C6—H6	0.93	C17—H17	0.93
N7—C8	1.363 (3)	C18—H18	0.93
N7—H7	0.82 (3)	O19—H19	0.86 (4)
C8—O9	1.230 (2)	O20—C21	1.429 (3)
C8—N10	1.341 (3)	C21—H21A	0.96
N10—C11	1.440 (3)	C21—H21B	0.96
N10—H10	0.86 (3)	C21—H21C	0.96
C11—C12	1.515 (4)	O22—C23	1.394 (4)
C11—H11A	0.97	C23—H23A	0.96
C11—H11B	0.97	C23—H23B	0.96
C12—C13	1.509 (3)	C23—H23C	0.96
			
C6—C1—C2	119.7 (2)	C13—C12—H12B	108.7
C6—C1—N7	123.2 (2)	C11—C12—H12B	108.7
C2—C1—N7	117.1 (2)	H12A—C12—H12B	107.6
O20—C2—C3	125.5 (2)	C18—C13—C14	116.88 (19)
O20—C2—C1	115.2 (2)	C18—C13—C12	122.3 (2)
C3—C2—C1	119.3 (2)	C14—C13—C12	120.8 (2)
C4—C3—C2	120.7 (2)	C13—C14—C15	121.7 (2)
C4—C3—H3	119.7	C13—C14—H14	119.1
C2—C3—H3	119.7	C15—C14—H14	119.1
C3—C4—C5	120.5 (3)	C16—C15—C14	120.3 (2)
C3—C4—H4	119.7	C16—C15—H15	119.8
C5—C4—H4	119.7	C14—C15—H15	119.8
O22—C5—C4	116.1 (2)	C17—C16—C15	119.08 (19)
O22—C5—C6	123.9 (2)	C17—C16—O19	122.8 (2)
C4—C5—C6	120.0 (2)	C15—C16—O19	118.1 (2)
C1—C6—C5	119.8 (2)	C16—C17—C18	119.7 (2)
C1—C6—H6	120.1	C16—C17—H17	120.1
C5—C6—H6	120.1	C18—C17—H17	120.1
C8—N7—C1	127.99 (19)	C13—C18—C17	122.2 (2)
C8—N7—H7	118.4 (18)	C13—C18—H18	118.9
C1—N7—H7	113.4 (18)	C17—C18—H18	118.9
O9—C8—N10	122.0 (2)	C16—O19—H19	110 (2)
O9—C8—N7	122.9 (2)	C2—O20—C21	117.5 (3)
N10—C8—N7	115.05 (18)	O20—C21—H21A	109.5
C8—N10—C11	122.76 (19)	O20—C21—H21B	109.5
C8—N10—H10	118.8 (17)	H21A—C21—H21B	109.5
C11—N10—H10	118.4 (17)	O20—C21—H21C	109.5
N10—C11—C12	114.0 (2)	H21A—C21—H21C	109.5
N10—C11—H11A	108.8	H21B—C21—H21C	109.5
C12—C11—H11A	108.8	C5—O22—C23	118.7 (2)
N10—C11—H11B	108.8	O22—C23—H23A	109.5
C12—C11—H11B	108.8	O22—C23—H23B	109.5
H11A—C11—H11B	107.7	H23A—C23—H23B	109.5
C13—C12—C11	114.19 (19)	O22—C23—H23C	109.5
C13—C12—H12A	108.7	H23A—C23—H23C	109.5
C11—C12—H12A	108.7	H23B—C23—H23C	109.5

### Hydrogen-bond geometry (Å, °)

**Table:**

*D*—H···*A*	*D*—H	H···*A*	*D*···*A*	*D*—H···*A*
N7—H7···O20	0.82 (3)	2.23 (2)	2.617 (3)	109 (2)
N7—H7···O19^i^	0.82 (3)	2.48 (3)	3.182 (3)	144 (2)
N10—H10···O19^i^	0.86 (3)	2.23 (3)	3.005 (3)	150 (2)
O19—H19···O9^ii^	0.86 (4)	1.80 (4)	2.654 (3)	172 (4)

Symmetry codes: (i) −*x*+1, −*y*+1, −*z*+1; (ii) *x*+1/2, −*y*+3/2, *z*+1/2.
